# British Hospitals Association

**Published:** 1921-03-05

**Authors:** 


### British Hospitals Association.

### FORMATION OF EASTERN REGIONAL COMMITTEE.

A well-attended meeting of representatives of voluntary hospitals in Norfolk, Suffolk, Cambridge and Huntingdonshire was held at the Norfolk and Norwich Hospital, Norwich, on Friday, February 25. Mr. R. Gurney, Chairman of the Norfolk and Norwich Hospital, explained the objects of the Association, and the advantages to be gained by the formation of a Regional Committee. It was unanimously decided that such a Regional Committee should be formed for East Anglia, consisting of two representatives each from the Norfolk and Norwich, East Suffolk and Ipswich, and Addenbrooke's Hospitals, and one representative from each of the smaller hospitals in the area affiliated to the Association. Mr. R. Gurney was unanimously elected Chairman for the ensuing year, and Mr. Frank Inch, Secretary of the Norfolk and Norwich Hospital, Hon. Secretary. It was agreed to hold the next meeting of the Committee at Norwich on March 18, when the representative of the Committee upon the Council of the Association will be appointed, and the question of being represented at the Government inquiry into the position of voluntary hospitals will be considered.

